# Distinct serum and cerebrospinal fluid cytokine and chemokine profiles in autoantibody-associated demyelinating diseases

**DOI:** 10.1177/2055217319848463

**Published:** 2019-05-15

**Authors:** Livia S Hofer, Sara Mariotto, Sebastian Wurth, Sergio Ferrari, Chiara R Mancinelli, Rachele Delogu, Salvatore Monaco, Alberto Gajofatto, Carmen Schwaiger, Kevin Rostasy, Florian Deisenhammer, Romana Höftberger, Thomas Berger, Markus Reindl

**Affiliations:** Clinical Department of Neurology, Medical University of Innsbruck, Austria; Department of Neuroscience, Biomedicine and Movement Sciences, University of Verona, Italy; Clinical Department of Neurology, Medical University of Innsbruck, Austria; Department of Neuroscience, Biomedicine and Movement Sciences, University of Verona, Italy; Multiple Sclerosis Centre, Spedali Civili di Brescia, Italy; Department of Clinical and Experimental Medicine, University of Sassari, Italy; Department of Neuroscience, Biomedicine and Movement Sciences, University of Verona, Italy; Department of Neuroscience, Biomedicine and Movement Sciences, University of Verona, Italy; Institute of Neurology, Medical University of Vienna, Austria; Paediatric Neurology, Witten/Herdecke University, Germany; Clinical Department of Neurology, Medical University of Innsbruck, Austria; Institute of Neurology, Medical University of Vienna, Austria; Department of Neurology, Medical University of Vienna, Austria; Clinical Department of Neurology, Medical University of Innsbruck, Austria

**Keywords:** Demyelinating diseases, aquaporin-4 antibody, myelin-oligodendrocyte-glycoprotein antibody, multiple sclerosis, cytokines, chemokines

## Abstract

**Background:**

Demyelinating diseases of the central nervous system associated with autoantibodies against aquaporin-4 and myelin-oligodendrocyte-glycoprotein are mediated by different immunopathological mechanisms compared to multiple sclerosis.

**Objective:**

The purpose of this study was to evaluate serum and cerebrospinal fluid cytokine/chemokine profiles in patients with autoantibodies against aquaporin-4 or autoantibodies against myelin-oligodendrocyte-glycoprotein-associated demyelination compared to multiple sclerosis and autoimmune encephalitis.

**Methods:**

Serum and cerebrospinal fluid cytokine/chemokine levels were analysed using Procartaplex Multiplex Immunoassays. First, we analysed a panel of 32 cytokines/chemokines in a discovery group (nine aquaporin-4-antibody seropositive, nine myelin oligodendrocyte glycoprotein-antibody seropositive, eight encephalitis, 10 multiple sclerosis). Significantly dysregulated cytokines/chemokines were validated in a second cohort (11 aquaporin-4-antibody seropositive, 18 myelin oligodendrocyte glycoprotein-antibody seropositive, 18 encephalitis, 33 multiple sclerosis).

**Results:**

We found 11 significantly altered cytokines/chemokines in cerebrospinal fluid and serum samples in the discovery group (a proliferation-inducing ligand, fractalkine=CX3CL1, growth-regulated oncogene-α, interleukin-1 receptor antagonist, interleukin-6, interleukin-8=CXCL8, interleukin-10, interleukin-21, interferon-ɣ-induced protein-10=CXCL10, monokine induced by interferon-ɣ=CXCL9, macrophage inflammatory protein-1ß=CCL4). Most of these cytokines/chemokines were up-regulated in autoantibodies against aquaporin-4 or autoantibodies against myelin-oligodendrocyte-glycoprotein positive patients compared to multiple sclerosis. We confirmed these results for cerebrospinal fluid interleukin-6 and serum interleukin-8, growth-regulated oncogene-α, a proliferation-inducing ligand and macrophage inflammatory protein-1β in the validation set. Receiver-operating characteristic analysis revealed increased levels of cerebrospinal fluid interleukin-6, serum interleukin-8 and growth-regulated oncogene-α in most patients with autoantibody-associated neurological diseases.

**Conclusion:**

This study suggests that distinctive cerebrospinal fluid and serum cytokine/chemokine profiles are associated with autoantibody-mediated demyelination, but not with multiple sclerosis.

## Introduction

Autoimmune diseases of the central nervous system (CNS) including aquaporin-4-antibody (AQP4-Ab) seropositive neuromyelitis optica spectrum disorders (NMOSDs), myelin oligodendrocyte glycoprotein-antibody (MOG-Ab) disease (AQP4-Ab seronegative NMOSD, optic neuritis (ON), transverse myelitis (TM), encephalitis and acute disseminated encephalomyelitis (ADEM)), anti-*N*-methyl-*D*-aspartate receptor-antibody (NMDAR-Ab) encephalitis and multiple sclerosis (MS) are characterised by complex interactions between the innate and adaptive immune system.^1–4^ Over the last years, these non-infectious inflammatory diseases have been the target of extensive research on aetiopathogenic mechanisms and on diagnostic aids. Autoantibodies targeting the astrocytic water channel aquaporin-4 (AQP4) have emerged as a highly sensitive and specific biomarker for the differentiation of NMOSD from MS, which is a crucial issue for an appropriate therapeutic choice.^5^ In fact, several MS treatments, such as interferon (IFN)-β, natalizumab and fingolimod, could lead to exacerbation of the NMOSD disease course, supporting the idea that NMOSD is distinct from MS and that NMOSD is dominated by humoral mechanisms. However, not all patients presenting with clinical features suggestive of a NMOSD disease phenotype are seropositive for AQP4-Ab and up to 50% of those patients are seropositive for MOG-Ab.^3^ Despite overlapping clinical presentations, multiple lines of data including (a) a lower proportion of females, (b) higher predominance of a monophasic disease course and (c) better functional recovery and steroid responsiveness in MOG-Ab positive patients indicate that AQP4-Ab and MOG-Ab diseases might have a different underlying pathogenesis and are distinct demyelinating conditions.^3^ Along these lines, specific molecular profiles may give some cues to the pathogenesis and aetiology of CNS inflammation useful for stratifying different clinical conditions and guiding novel treatment strategies.

Previous reports demonstrated that T helper (Th)-17- and Th2-associated cytokines are up-regulated in patients with NMOSD, whereas MS is primarily a Th1-dominant disease suggesting that cytokine/cheokine profiles in NMOSD are different from MS.^6–8^ A recent study underpins this assumption by showing that the CSF cytokine profile in MOG-Ab disease is similar to AQP4-immunoglobulin (Ig)-G positive NMOSD but clearly distinct from MS.^9^ Among the Th17-axis cytokines, specific attention has been recently devoted to interleukin (IL)-6, which is involved in tissue regeneration, inflammation and defence against pathogens with a central role in CNS neuroinflammatory pathways.^10^ High levels of IL-6 have been particularly reported in the CSF of AQP4-Ab NMOSD patients, suggesting that this biomarker might be a predictor of disease severity.^6^^,^^11^^,^^12^ Increased IL-6 levels in the CSF have been also described in MOG-Ab disease and in subjects with various forms of viral or autoimmune encephalitis, in particular in those with NMDAR-Ab.^13–15^ In light of these observations, blockade of IL-6 receptor signalling has been suggested as a possible therapeutic strategy in severe cases of AQP4-Ab and MOG-Ab disease as well as autoimmune encephalitis refractory to conventional immunotherapies.^16–21^

Therefore, the main aim of this study was to evaluate cytokine/chemokine profiles, with a particular focus on IL-6, in paired serum and CSF samples of patients in the acute phase of AQP4-Ab and MOG-Ab-associated CNS demyelinating diseases in comparison to MS and anti-NMDAR encephalitis and to unravel their utility as biomarkers to distinguish Ab-mediated conditions from MS.

## Material and methods

### Study subjects and clinical data

**Figure 1.**
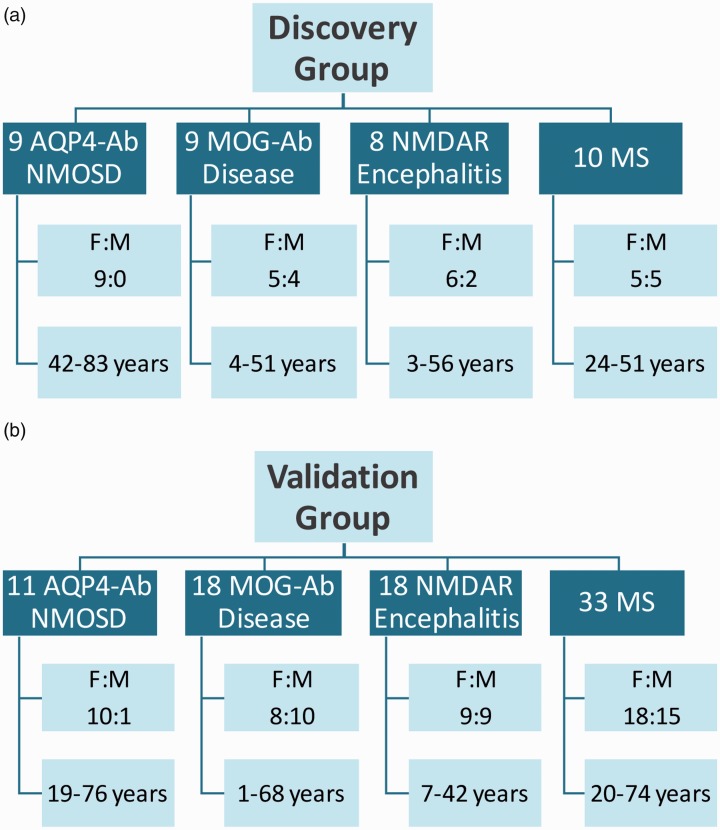
Demographic characteristics of participants from the (a) discovery and (b) validation group. Ab: antibody; AQP-4: aquaporin-4; F: female; M: male; MOG: myelin oligodendrocyte glycoprotein; MS: multiple sclerosis; NMDAR: *N*-methyl-*D*-aspartate receptor; NMOSD: neuromyelitis optica spectrum disorder.

Serum and CSF samples were collected and stored at three diagnostic centres (Neuropathology Laboratory, University Hospital of Verona, Italy; Clinical Department of Neurology, Medical University of Innsbruck, Austria; Institute of Neurology, Medical University of Vienna, Austria) between 2009–2018. This study was approved by the Ethical Committee of the University of Verona (study number 1052CESC), the Medical University of Innsbruck (study numbers AM3041A and AM4059) and the Medical University of Vienna (study number EK1123/2015). All available samples from patients from the three diagnostic centres in Austria (Innsbruck, Vienna) and Italy (Verona) were included. All samples were collected during the diagnostic uptake (median disease duration 2.5 months, range 0–264 months) in the acute phase. Patients or their caregivers gave written informed consent to diagnostic procedures and biological sample storage. The discovery group was represented by nine AQP4-Ab patients (nine females, age 42–83 years), nine subjects with MOG-Ab disease (five females and four males, age 4–51 years), eight anti-NMDAR encephalitis cases (six females and two males, age 3–56 years) and 10 MS patients (five females and five males, age 24–51 years) (Figure 1(a)). The validation group consisted of 11 AQP4-Ab patients (10 females and one male, age 19–76 years), 18 subjects with MOG-Ab disease (eight females and 10 males, age 1–68 years), 18 anti-NMDAR encephalitis cases (nine females and nine males, age 7–42 years) and 33 MS patients (18 females and 15 males, age 20–74 years) (Figure 1(b)).

All patients with CNS disorders were classified according to specific diagnostic criteria into four categories: (a) NMOSD, (b) MS, (c) other demyelinating conditions including ADEM, idiopathic ON, idiopathic myelitis and idiopathic inflammatory disorders and (d) anti-NMDAR-encephalitis.^5,22–24^ Idiopathic ON and/or myelitis were defined as one or more episodes of acute/subacute optic neuropathy and/or myelopathy of inflammatory origin (based on clinical, radiological and/or CSF evidence) not fulfilling diagnostic criteria for MS, NMOSD and ADEM and not attributable to other causes. The cohort was mainly composed of adults. Of all included patients (116) from both cohorts 11/27 (41%) MOG-Ab positive cases and 7/26 (27%) patients with NMDAR-Ab were children, whereas the AQP4-Ab and MS groups only included adults.

### AQP4-Ab, MOG-Ab and NMDAR-Ab detection assays

Serum AQP4-Ab and serum/CSF NMDAR-Ab were analysed using commercially available cell-based assays (Euroimmun, Luebeck, Germany) according to the manufacturer's instructions or by live cell- or tissue-based assays.^25^

Serum MOG-Ab was analysed using recombinant live cell-based immunofluorescence assay with HEK293A cells transfected with full-length MOG (human MOG alpha-1 enhanced green fluorescent protein (EGFP) fusion protein), as described previously.^25^ Sera were tested at dilutions of 1:20 and 1:40 and MOG-Ab positivity was titrated with serial dilutions with a threshold of 1:160 to define MOG-Ab positivity.

### Cytokine and chemokine immunoassays

Levels of cytokines/chemokines in serum and CSF pairs of patients of the discovery group were determined using a commercially available custom 32-plex Procartaplex Multiplex Immunoassay (Thermo Fisher Scientific, Waltham, MA, USA; cat.#: PPX-32-MXCE33Y) according to the manufacturer's instructions. This magnetic bead assay is based on the Luminex xMAP technology, which enables the simultaneous detection and quantitation of multiple secreted cytokines/chemokines, namely: a proliferation-inducing ligand (APRIL), B cell activating factor (BAFF), B lymphocyte chemoattractant (BLC) or CXC-chemokine ligand (CXCL)-13, CD40 ligand (CD40L), eotaxin or CC-chemokine ligand (CCL)-11, fractalkine or CX3C-chemokine ligand (CX3CL)-1, granulocyte colony-stimulating factor (G-CSF) or CSF-3, granulocyte macrophage colony-stimulating factor (GM-CSF), growth-regulated oncogene (GRO)-α or KC/CXCL1, IFN-α, IFN-ɣ, IL-1β, IL-1 receptor antagonist (RA), IL-2, IL-4, IL-5, IL-6, IL-8 (CXCL8), IL-10, IL-12p70, IL-13, IL-17A or cytotoxic T-lymphocyte-associated protein (CTLA)-8, IL-21, IL-23, IFN-ɣ-induced protein (IP)-10 or CXCL10, monocyte chemoattractant protein (MCP)-1 or CCL2, monokine induced by interferon-ɣ (MIG) or CXCL9, macrophage inflammatory protein (MIP)-1α or CCL3, MIP-1β (CCL4), MIP-3α (CCL20), stromal cell-derived factor (SDF)-1α, tumour necrosis factor (TNF)-α (Supplementary Material Table 1). Next, levels of cytokines/chemokines in serum and CSF pairs of 80 patients of the validation group were investigated using Procartaplex Human Basic Kits (Thermo Fisher Scientific, Waltham, Massachusetts, USA; cat.#: EPX010-10420-901) combining six and five different cytokine/chemokine beads that were found to be significantly dysregulated in the discovery group, namely APRIL, GRO-α, MIP-1β, IL-1RA, IL-6, IL-8 (Standard-Mix A Lot#162779101, Standard-Mix B Lot#149689000. Standard-Mix K Lot#:124478000) and fractalkine, IL-10, IL-21, IP-10, MIG (Standard-Mix A Lot#180560101, Standard-Mix B Lot#182229101, Standard-Mix D Lot#140146301), respectively (Supplementary Material Table 1).

Multiplex assays were performed according to the manufacturer's instructions. Briefly, magnetic beads at working concentration were added into each well of the kit-provided black 96-well flat bottom plates. After washing of the beads and removal of any residual liquid, samples, standards and blanks were added. For serum and CSF pairs, 25 μl of the kit-provided 1× Universal Assay Buffer was added to each well followed by 25 μl four-fold serial diluted standards or undiluted samples into dedicated wells. For wells designated as blanks, additional 25 μl Buffer was added. The plate was incubated for 30 min with agitation at 300 rounds per min (rpm) at room temperature (RT) and protection from light exposure. Afterwards, the plate was transferred on a level surface at 4°C. After overnight incubation, the plate was again shaken at 300 rpm for 30 min at RT and then the magnetic beads were washed. Next, 25 μl of 1× Detection Antibody Mixture was added to each well and incubated for 30 min with agitation at 300 rpm at RT and light protection. After washing of magnetic beads, 50 μl of streptavidin-phycoerythrin (SAPE) solution was added and incubated for 30 min as described above. After another washing step, the test plate was prepared for analysis on a Luminex instrument by adding 120 μl of Reading Buffer into each well and incubation with agitation at 300 rpm for five minutes at RT. Finally, the plate was analysed on the Luminex MAGPIX instrument (Software: xPonent 4.2). The cytokine/chemokine concentrations were calculated using the standard curve generated by the five-parameter logistic regression method. In samples where cytokines/chemokines were undetectable, the values of the detection limit were used for analyses.

### Statistical analysis

Statistical analyses were performed using IBM SPSS software (IBM Corp. Released 2012. IBM SPSS Statistics; Version 21.0. Armonk, New York, USA: IBM Corp.) and GraphPad Prism 7 (GraphPad Software, La Jolla, California, USA). The null hypothesis (H0) for the 32-plex discovery experiment was that none of the serum or CSF cytokines/chemokines were significantly different between the four groups. Overall significance was assessed using the nonparametric Kruskal Wallis test and *p*-values were adjusted for multiple comparisons using a false discovery rate (FDR) significance criterion of 10% based on the Benjamini-Hochberg correction. Between-group comparisons were calculated using Dunn’s multiple comparison test. Eleven dysregulated cytokines/chemokines were then confirmed in the validation group and statistical analysis was done as described above. Receiver-operating characteristic (ROC) analysis was performed on pooled samples to determine cut-off values for cytokines/chemokines dysregulated in both data sets. Correlation coefficients of the five significantly dysregulated cytokines/chemokines between CSF and serum were evaluated using the Spearman’s rho correlation test.

## Results

### Significantly altered cytokine and chemokine profiles in CSF and serum samples from patients with neuroinflammatory disorders

**Figure 2.**
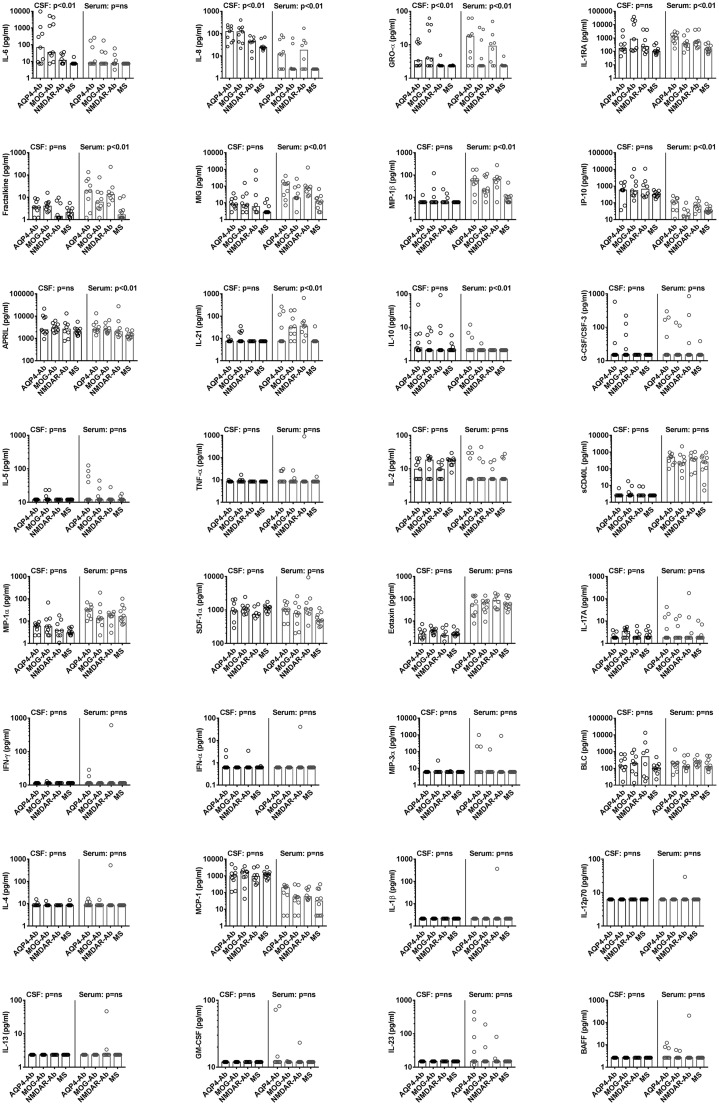
Scatter dot plots of cytokines and chemokines in cerebrospinal fluid (CSF) and serum of the discovery set. Individual values for aquaporin-4-antibody (AQP4-Ab), myelin oligodendrocyte glycoprotein-antibody (MOG-Ab), anti-N-methyl-D-aspartate receptor-antibody (NMDAR-Ab) and multiple sclerosis (MS) patients are shown as circles and medians are shown as bars. Cytokines and chemokines are organised according to their *p*-values. Overall significance was assessed using the nonparametric Kruskal-Wallis test and *p*-values were adjusted for multiple comparisons using a false discovery rate (FDR) significance criterion of 10% based on the Benjamini-Hochberg correction. Between group comparisons were calculated using Dunn’s multiple comparison test. Significant changes in the CSF (AQP4-Ab and MOG-Ab versus MS) and serum (AQP4-Ab, MOG-Ab and NMDAR-Ab versus MS) are shown in the graphs.Ab: antibody; AQP-4: aquaporin-4; APRIL: a proliferation-inducing ligand; BAFF: B cell activating factor; BLC: B lymphocyte chemoattractant; G-CSF: granulocyte colony-stimulating factor; GM-CSF: granulocyte macrophage colony-stimulating factor; GRO: growth-regulated oncogene; IFN: interferon; IL: interleukin; IP: interferon-ɣ-induced protein; MCP: monocyte chemoattractant protein; MIG: monokine induced by interferon-ɣ; MIP: macrophage inflammatory protein; MOG: myelin oligodendrocyte glycoprotein; MS: multiple sclerosis; NMDAR: *N*-methyl-*D*-aspartate receptor; ns: not significant; SDF: stromal cell-derived factor; TNF: tumour necrosis factor.

First, we have analysed the levels of 32 different cytokines/chemokines in paired CSF and serum samples from the discovery group (Figure 1(a), Supplementary Material Table 1). In this discovery cohort the levels of three cytokines/chemokines (IL-6, IL-8 and GRO-α) were significantly different between groups in the CSF and 10 cytokines/chemokines (IL-1RA, fractalkine, MIG, MIP-1β, IP-10, APRIL, IL-21, IL-8, IL-10 and GRO-α; cytokines/chemokines organised according to their *p*-values) were significantly different in the serum (Figure 2, Supplementary Material Material Figure 1).

**Figure 3.**
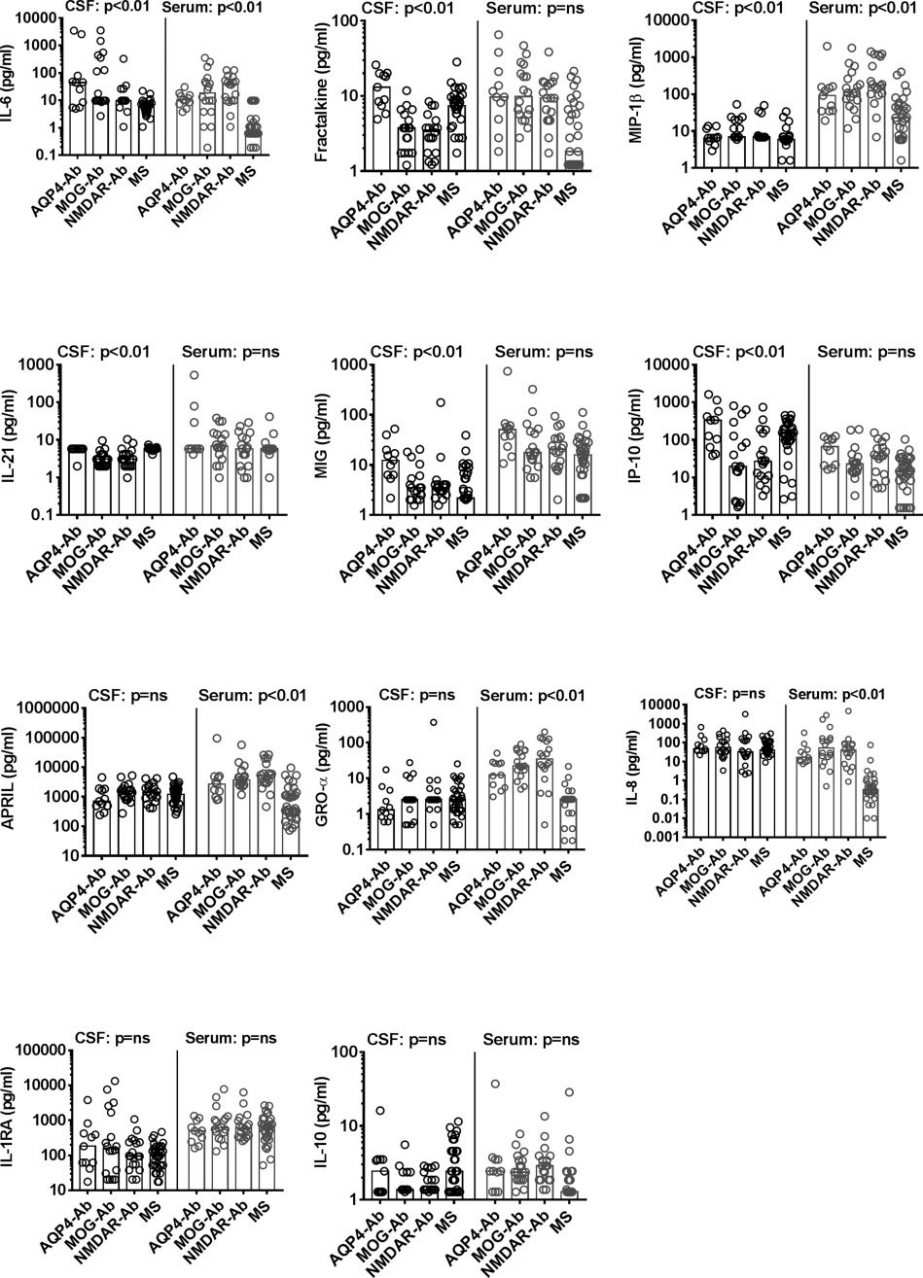
Scatter dot plots of cytokines and chemokines in cerebrospinal fluid (CSF) and serum of the validation set. Individual values for aquaporin-4-antibody (AQP4-Ab), myelin oligodendrocyte glycoprotein-antibody (MOG-Ab), anti-N-methyl-D-aspartate receptor-antibody (NMDAR-Ab) and multiple sclerosis (MS) patients are shown as circles and medians are shown as bars. Cytokines and chemokines are organised according to their *p*-values. Overall significance was assessed using the nonparametric Kruskal Wallis test and *p*-values were adjusted for multiple comparisons using a false discovery rate (FDR) significance criterion of 10% based on the Benjamini-Hochberg correction. Between group comparisons were calculated using Dunn’s multiple comparison test. Significant changes in the CSF (AQP4-Ab and MOG-Ab versus MS) and serum (AQP4-Ab, MOG-Ab and NMDAR-Ab versus MS) are shown in the graphs.Ab: antibody; AQP-4: aquaporin-4; GRO: growth-regulated oncogene; IL: interleukin; IP: interferon-ɣ-induced protein; MCP: monocyte chemoattractant protein; MIG: monokine induced by interferon-ɣ; MIP: macrophage inflammatory protein; MOG: myelin oligodendrocyte glycoprotein; MS: multiple sclerosis; NMDAR: *N*-methyl-*D*-aspartate receptor; ns: not significant.

In a next step, the 11 most significantly dysregulated cytokines/chemokines (APRIL, fractalkine, GRO-α, MIG, MIP-1β, IL-1RA, IL-6, IL-8, IL-10, IL-21 and IP-10) were validated in paired CSF and serum samples from the validation group (Figure 1(b), Supplementary Material Table 1). In this confirmatory analysis the levels of six cytokines/chemokines (IL-6, fractalkine, MIP-1β, IL-21, MIG and IP-10) were significantly different between groups in the CSF and five cytokines/chemokines (IL-8, GRO-α, IL-6, APRIL and MIP-1β; cytokines/chemokines organised according to their *p*-values) were significantly different in the serum (Figure 3, Supplementary Material Figure 1). Thus, we could confirm the results from the discovery experiment for CSF IL-6 and serum IL-8, GRO-α, APRIL and MIP-1β.

### CSF IL-6 and serum IL-8, GRO-α, APRIL and MIP-1β are predictors of antibody-mediated conditions

**Figure 4.**
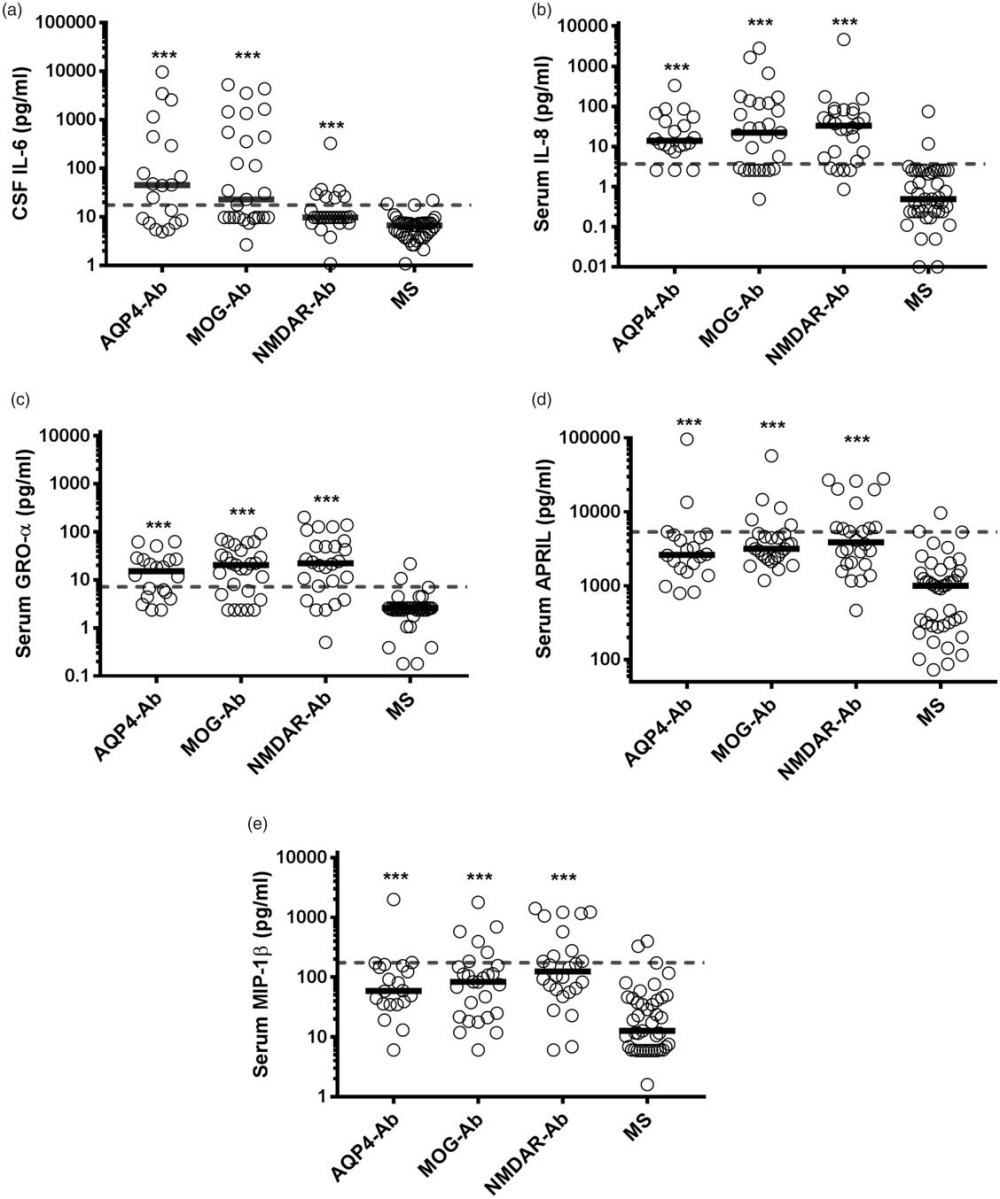
Dysregulated cytokines and chemokines confirmed in both cohorts. Pooled individual values for both cohorts are shown as circles and medians are shown as bars. Significance of group differences was analysed by the Kruskal Wallis test and significant differences (*p*-value <0.001) are shown by the asterisks.The grey dashed lines represent the cut-off value of CSF IL-6 (a) and of serum IL-8 (b), GRO-α (d), APRIL (d) and MIP-1β (d) to discriminate AQP4-Ab, MOG-Ab and NMDAR-Ab from MS patients with 95% specificity. Ab: antibody; APRIL: a proliferation-inducing ligand; AQP-4: aquaporin-4; CSF: cerebrospinal fluid; GRO: growth-regulated oncogene; IL: interleukin; MIP: macrophage inflammatory protein; MOG: myelin oligodendrocyte glycoprotein; MS: multiple sclerosis; NMDAR: *N*-methyl-*D*-aspartate receptor.

In a final step we have pooled and analysed the data from both experiments for the five significantly dysregulated cytokines/chemokines in either the CSF or serum in order to evaluate, which of the CSF and/or serum cytokines/chemokines were the best predictors of antibody-mediated conditions. Using ROC analysis, we calculated area under the curve (AUC) values and determined cut-off values of CSF IL-6 and of serum IL-8, GRO-α, APRIL and MIP-1β to discriminate AQP4-Ab, MOG-Ab and NMDAR-Ab from MS patients with 95% specificity (Figure 4, Table 1) Serum IL-8 level ≥3.7 pg/ml had the highest sensitivity (77%) for AQP4-Ab and MOG-Ab-associated demyelinating diseases, followed by serum GRO-α (cut-off ≥7.2 pg/ml, sensitivity 70%) and CSF IL-6 (cut-off ≥17.6 pg/ml, sensitivity 47%). When combining the cut-off values for serum IL-8 and GRO-α, none of the MS patients, but 60% of AQP4-Ab positive patients and 63% of MOG-Ab positive patients had increased values. However, this increased serum cytokine/chemokine response was not specific for AQP4-Ab and MOG-Ab-associated demyelinating diseases, but also seen in anti-NMDAR encephalitis (77%) indicating a common inflammatory response in antibody (Ab)-associated neurological autoimmune diseases, which is not observed in MS.

**Table 1.** Receiver-operating characteristic (ROC) analysis of dysregulated cytokines and chemokines.

Variable	Area under the curve (95% CI)	*p*-Value	Cut-off (pg/ml)	AQP4-Ab	MOG-Ab	NMDAR-Ab	MS
CSF IL-6	0.887 (0.818–0.956)	<0.001	17.6	12/20 (60%)	15/27 (56%)	7/26 (27%)	2/43 (5%)
Serum IL-8	0.936 (0.886–0.989	<0.001	3.7	17/20 (85%)	18/27 (67%)	21/26 (81%)	2/43 (5%)
Serum GROα	0.874 (0.799–0.950)	<0.001	7.2	12/20 (60%)	19/27 (70%)	20/26 (77%)	2/43 (5%)
Serum APRIL	0.857 (0.775–0.939)	<0.001	5339.6	3/20 (15%)	5/27 (18%)	12/26 (46%)	2/43 (5%)
Serum MIP-1ß	0.791 (0.697–0.885)	<0.001	174.2	2/20 (10%)	6/27 (22%)	10/26 (38%)	2/43 (5%)

Ab: antibody; APRIL: a proliferation-inducing ligand; AQP-4: aquaporin-4; CI: confidence interval; CSF: cerebrospinal fluid; GRO: growth-regulated oncogene; IL: interleukin; MIP: macrophage inflammatory protein; MOG: myelin oligodendrocyte glycoprotein; MS: multiple sclerosis; NMDAR: *N*-methyl-*D*-aspartate receptor.

Moreover, correlations of the five significantly dysregulated cytokines/chemokines between CSF and serum were evaluated (Table 2). In MS patients, significant correlations were found for the Th17-related cytokine IL-6 and for the B cell-related cytokine APRIL. In MOG-Ab positive patients, significant correlations were observed for the broad spectrum-related chemokine MIP-1ß.

**Table 2.** Correlation of dysregulated cytokines and chemokines between cerebrospinal fluid (CSF) and serum.

Variable	Overall (*n*=116) *r*_s_	AQP4-Ab (*n*=20) *r*_s_	MOG-Ab (*n*=27) *r*_s_	NMDAR-Ab (*n*=26) *r*_s_	MS (*n*=43) *r*_s_
IL-6^a^	0.510^b^	–0.200	0.188	0.308	0.465^b^
IL-8^a^	0.126	–0.078	0.180	–0.257	0.061
GRO-α^a^	0.048	0.119	–0.199	0.031	0.000
APRIL^a^	0.201^c^	0.047	–0.088	0.006	0.447^b^
MIP-1ß^a^	0.435^b^	0.077	0.535^b^	0.213	0.046

Ab: antibody; APRIL: a proliferation-inducing ligand; AQP-4: aquaporin-4; GRO: growth-regulated oncogene; IL: interleukin; MIP: macrophage inflammatory protein; MOG: myelin oligodendrocyte glycoprotein; MS: multiple sclerosis; NMDAR: *N*-methyl-*D*-aspartate receptor.

^a^Correlation coefficients (*r*_s_) were analysed using Spearman’s rho correlation test.

^b^Significant correlations *p*≤0.01 and ^c^significant correlations *p*≤0.05 of dysregulated cytokines and chemokines between CSF and serum.

Finally, we have analysed possible clinical associations for these cut-off values. Due to the retrospective character of our study, the data set is incomplete and only allows an explanatory analysis. As can be seen from Supplementary Material Tables 2–4 there were no statistically significant differences for age, sex, clinical presentation or diagnosis, neither overall nor for the MOG-Ab and AQP4-Ab subgroups. There was a tendency (*p*<0.1) for a higher frequency of myelitis in patients with increased CSF IL-6 levels in both subgroups and for increased serum GRO-α levels in MOG-Ab positive children. CSF cell counts and CSF protein levels were significantly increased in patients with increased CSF IL-6 levels, overall and in the MOG-Ab subgroup.

## Discussion

It has been suggested that cytokine/chemokine profiles may reflect distinct immunopathological processes between Ab-associated conditions and MS. They might not only be important for a better understanding of the pathophysiology of these syndromes, but may also prove as useful biomarkers for stratification of patients, which is a prerequisite for the appropriate treatment choice. We found significantly increased levels of the Th17-related cytokine IL-6 in the CSF as well as IL-8 (Th17-related), APRIL (B cell-related), GRO-α and MIP-1β (broad spectrum-related) in the serum of patients with AQP4-Ab, MOG-Ab and NMDAR-Ab as compared to MS, which we could confirm in a validation set. Additionally, ROC analysis from the combined discovery and validation cohort revealed significantly increased levels of those five cytokines/chemokines in Ab-mediated conditions suggesting that they are predictors of Ab-associated diseases clearly distinctive from MS. Although we did not find significantly altered serum IL-6 levels in the discovery set, serum IL-6 levels were significantly increased in the validation set but also in pooled data from both experiments. In this regard, IL-6, the key polarising cytokine of Th17 cells, has pleiotropic effects mediated through the transmembrane protein gp130 including modulation of nociceptive neurons associated with pain, T and B cell activation, Th17 differentiation, immunoglobulin synthesis as well as plasmablast survival.^10,26^ Thus, these findings might be explained by the critical IL-6 involvement in the activation and maintenance of humoral responses and might also indicate active cooperation of Th17 and B cells in the immunopathogenicity of Ab-mediated inflammatory disorders.^26^ Significantly higher levels of APRIL in the serum of Ab-mediated conditions, which is a crucial plasmablast factor regulating B cell survival, differentiation and class switching underpin this interrelation.^27^ Additionally, up-regulation of Th17-related (IL-8) and broad spectrum-related chemokines (GRO-α, MIP-1β) with potent chemoattractant properties in the serum of patients with Ab-associated conditions might add to these pathological processes by recruiting inflammatory cells such as neutrophils to targeted sites.^28^ In this context, as proposed in AQP4-Ab positive cases, IL-6 signalling might facilitate Ab accumulation in the target sites promoting Ab-mediated autoimmunity, and might also cause a decrease in blood brain barrier (BBB) function, an increase in chemokine production resulting in enhanced leukocyte transmigration.^29,30^ While controversial data have been reported on serum IL-6 levels in NMOSD patients, more recent findings confirm the significant elevation of CSF IL-6 concentration in this condition, suggesting that this biomarker might be potentially used to differentiate NMOSD from MS.^11,31,32^ Moreover, up to now, only a few studies have analysed IL-6 levels in the biological fluids of subjects with conditions related to MOG-Ab and all focused on CSF values.^9,15,33^ According to these reports, MOG-Ab positive patients seem to have higher CSF IL-6 levels than seronegative patients, which also correlates with MOG-Ab titres.^15,33^ Currently, tocilizumab, a humanised monoclonal antibody targeting the IL-6 receptor (IL-6R) and approved for the treatment of rheumatoid arthritis has been successfully used as off-label therapy to effectively reduce relapse rate and disability, neuropathic pain and general fatigue in AQP4-Ab positive NMOSD and is suggested as a third-line treatment in severe and treatment-resistant cases.^34^ But also recent reports in MOG-Ab-associated diseases and autoimmune encephalitis have shown promising effects in patients having failed various immunotherapies including rituximab, which is a B-cell depleting therapy.^17,20,21^ Similar to disease exacerbation of NMOSD patients in response to MS treatments, it has been reported that anti-IL-6R blockade might pose a risk to cause MS-like demyelinating disorders.^35^ However, in line with the immunological and biological differences in Ab-mediated conditions and MS, which we could also observe in our study, this might rather relate to different underlying pathophysiological processes involved in these diseases. These data also suggest that the IL-6 pathway may be spared in MS and underline the relevance of different pathogenic mechanisms in inducing the associated immune response. It might seem surprising to find a similar cytokine profile for Ab-associated demyelinating diseases and anti-NMDAR-Ab encephalitis, despite different clinical courses and antigenic targets. These conditions are all characterised by a specific contribution of humoral responses and autoantibody production. A possible explanation could be that the immunological processes for the generation of autoantibodies are virtually the same, while the affected biological target determines the clinical phenotype.

The main limitation of our study is its retrospective character and the incomplete clinical documentation of included cases. Therefore, our study should be considered as a pilot study, which informs the scientific community about our results and fosters confirmatory prospective studies to establish novel biomarkers for Ab-associated demyelinating disorders.

In conclusion, we found a distinctive cytokine/chemokine profile in Ab-associated conditions compared to MS. Especially, IL-6 constitutes not only an important biomarker of Ab-mediated inflammatory CNS diseases but also represents an important therapeutic target in NMOSD. Furthermore, our observation that increased serum IL-8 and GRO-α were absent in MS patients, but present in 60% of AQP4-Ab positive patients and 63% of MOG-Ab positive patients might be helpful to establish novel diagnostic biomarkers for these syndromes.

## Supplemental Material

Supplemental Material1 - Supplemental material for Distinct serum and cerebrospinal fluid cytokine and chemokine profiles in autoantibody-associated demyelinating diseases Click here for additional data file. Supplemental material, Supplemental Material1 for Distinct serum and cerebrospinal fluid cytokine and chemokine profiles in autoantibody-associated demyelinating diseases by Livia S Hofer Clinical Department of Neurology, Medical University of Innsbruck, Austria Sara Mariotto Department of Neuroscience, Biomedicine and Movement Sciences, University of Verona, Italy Sebastian Wurth Clinical Department of Neurology, Medical University of Innsbruck, Austria Sergio Ferrari Department of Neuroscience, Biomedicine and Movement Sciences, University of Verona, Italy Chiara R Mancinelli Multiple Sclerosis Centre, Spedali Civili di Brescia, Italy Rachele Delogu Department of Clinical and Experimental Medicine, University of Sassari, Italy Salvatore Monaco Department of Neuroscience, Biomedicine and Movement Sciences, University of Verona, Italy Alberto Gajofatto Department of Neuroscience, Biomedicine and Movement Sciences, University of Verona, Italy Carmen Schwaiger Institute of Neurology, Medical University of Vienna, Austria Kevin Rostasy Paediatric Neurology, Witten/Herdecke University, Germany Florian Deisenhammer Clinical Department of Neurology, Medical University of Innsbruck, Austria Romana Höftberger Institute of Neurology, Medical University of Vienna, Austria Thomas Berger Department of Neurology, Medical University of Vienna, Austria Markus Reindl Clinical Department of Neurology, Medical University of Innsbruck, Austria in Multiple Sclerosis Journal—Experimental, Translational and Clinical

## Supplemental Material

Supplemental Material2 - Supplemental material for Distinct serum and cerebrospinal fluid cytokine and chemokine profiles in autoantibody-associated demyelinating diseases Click here for additional data file. Supplemental Material2 - Supplemental material for Distinct serum and cerebrospinal fluid cytokine and chemokine profiles in autoantibody-associated demyelinating diseases by Livia S Hofer Clinical Department of Neurology, Medical University of Innsbruck, Austria Sara Mariotto Department of Neuroscience, Biomedicine and Movement Sciences, University of Verona, Italy Sebastian Wurth Clinical Department of Neurology, Medical University of Innsbruck, Austria Sergio Ferrari Department of Neuroscience, Biomedicine and Movement Sciences, University of Verona, Italy Chiara R Mancinelli Multiple Sclerosis Centre, Spedali Civili di Brescia, Italy Rachele Delogu Department of Clinical and Experimental Medicine, University of Sassari, Italy Salvatore Monaco Department of Neuroscience, Biomedicine and Movement Sciences, University of Verona, Italy Alberto Gajofatto Department of Neuroscience, Biomedicine and Movement Sciences, University of Verona, Italy Carmen Schwaiger Institute of Neurology, Medical University of Vienna, Austria Kevin Rostasy Paediatric Neurology, Witten/Herdecke University, Germany Florian Deisenhammer Clinical Department of Neurology, Medical University of Innsbruck, Austria Romana Höftberger Institute of Neurology, Medical University of Vienna, Austria Thomas Berger Department of Neurology, Medical University of Vienna, Austria Markus Reindl Clinical Department of Neurology, Medical University of Innsbruck, Austria in Multiple Sclerosis Journal—Experimental, Translational and Clinical

## Supplemental Material

Supplemental Material3 - Supplemental material for Distinct serum and cerebrospinal fluid cytokine and chemokine profiles in autoantibody-associated demyelinating diseases Click here for additional data file. Supplemental material, Supplemental Material3 for Distinct serum and cerebrospinal fluid cytokine and chemokine profiles in autoantibody-associated demyelinating diseases by Livia S Hofer Clinical Department of Neurology, Medical University of Innsbruck, Austria Sara Mariotto Department of Neuroscience, Biomedicine and Movement Sciences, University of Verona, Italy Sebastian Wurth Clinical Department of Neurology, Medical University of Innsbruck, Austria Sergio Ferrari Department of Neuroscience, Biomedicine and Movement Sciences, University of Verona, Italy Chiara R Mancinelli Multiple Sclerosis Centre, Spedali Civili di Brescia, Italy RacheleDelogu Department of Clinical and Experimental Medicine, University of Sassari, Italy Monaco Salvatore Department of Neuroscience, Biomedicine and Movement Sciences, University of Verona, Italy Alberto Gajofatto Department of Neuroscience, Biomedicine and Movement Sciences, University of Verona, Italy Carmen Schwaiger Institute of Neurology, Medical University of Vienna, Austria Kevin Rostasy Paediatric Neurology, Witten/Herdecke University, Germany Florian Deisenhammer Clinical Department of Neurology, Medical University of Innsbruck, Austria Romana Höftberger Institute of Neurology, Medical University of Vienna, Austria Thomas Berger Department of Neurology, Medical University of Vienna, Austria Markus Reindl Clinical Department of Neurology, Medical University of Innsbruck, Austria in Multiple Sclerosis Journal—Experimental, Translational and Clinical

## Supplemental Material

Supplemental Material4 - Supplemental material for Distinct serum and cerebrospinal fluid cytokine and chemokine profiles in autoantibody-associated demyelinating diseases Click here for additional data file. Supplemental material, Supplemental Material4 for Distinct serum and cerebrospinal fluid cytokine and chemokine profiles in autoantibody-associated demyelinating diseases by Livia S Hofer Clinical Department of Neurology, Medical University of Innsbruck, Austria Sara Mariotto Department of Neuroscience, Biomedicine and Movement Sciences, University of Verona, Italy Sebastian Wurth Clinical Department of Neurology, Medical University of Innsbruck, Austria Sergio Ferrari Department of Neuroscience, Biomedicine and Movement Sciences, University of Verona, Italy Chiara R Mancinelli Multiple Sclerosis Centre, Spedali Civili di Brescia, Italy RacheleDelogu Department of Clinical and Experimental Medicine, University of Sassari, Italy Monaco Salvatore Department of Neuroscience, Biomedicine and Movement Sciences, University of Verona, Italy Alberto Gajofatto Department of Neuroscience, Biomedicine and Movement Sciences, University of Verona, Italy Carmen Schwaiger Institute of Neurology, Medical University of Vienna, Austria Kevin Rostasy Paediatric Neurology, Witten/Herdecke University, Germany Florian Deisenhammer Clinical Department of Neurology, Medical University of Innsbruck, Austria Romana Höftberger Institute of Neurology, Medical University of Vienna, Austria Thomas Berger Department of Neurology, Medical University of Vienna, Austria Markus Reindl Clinical Department of Neurology, Medical University of Innsbruck, Austria in Multiple Sclerosis Journal—Experimental, Translational and Clinical

## Supplemental Material

Supplemental Material5 - Supplemental material for Distinct serum and cerebrospinal fluid cytokine and chemokine profiles in autoantibody-associated demyelinating diseases Click here for additional data file. Supplemental material, Supplemental Material5 for Distinct serum and cerebrospinal fluid cytokine and chemokine profiles in autoantibody-associated demyelinating diseases by Livia S Hofer Clinical Department of Neurology, Medical University of Innsbruck, Austria Sara Mariotto Department of Neuroscience, Biomedicine and Movement Sciences, University of Verona, Italy Sebastian Wurth Clinical Department of Neurology, Medical University of Innsbruck, Austria Sergio Ferrari Department of Neuroscience, Biomedicine and Movement Sciences, University of Verona, Italy Chiara R Mancinelli Multiple Sclerosis Centre, Spedali Civili di Brescia, Italy RacheleDelogu Department of Clinical and Experimental Medicine, University of Sassari, Italy Monaco Salvatore Department of Neuroscience, Biomedicine and Movement Sciences, University of Verona, Italy Alberto Gajofatto Department of Neuroscience, Biomedicine and Movement Sciences, University of Verona, Italy Carmen Schwaiger Institute of Neurology, Medical University of Vienna, Austria Kevin Rostasy Paediatric Neurology, Witten/Herdecke University, Germany Florian Deisenhammer Clinical Department of Neurology, Medical University of Innsbruck, Austria Romana Höftberger Institute of Neurology, Medical University of Vienna, Austria Thomas Berger Department of Neurology, Medical University of Vienna, Austria Markus Reindl Clinical Department of Neurology, Medical University of Innsbruck, Austria in Multiple Sclerosis Journal—Experimental, Translational and Clinical
