# Supplementary material for: Distinct serum and cerebrospinal fluid cytokine and chemokine profiles in autoantibody-associated demyelinating diseases
Source: Mult Scler J Exp Transl Clin. 2019 May 15;5(2):2055217319848463. doi: 10.1177/2055217319848463 (PMC6537078; doi:10.1177/2055217319848463)
Supplement: Supplemental Material1 - Supplemental material for Distinct serum and cerebrospinal fluid cytokine and chemokine profiles in autoantibody-associated demyelinating diseases [file Supplemental_Material1.pdf]

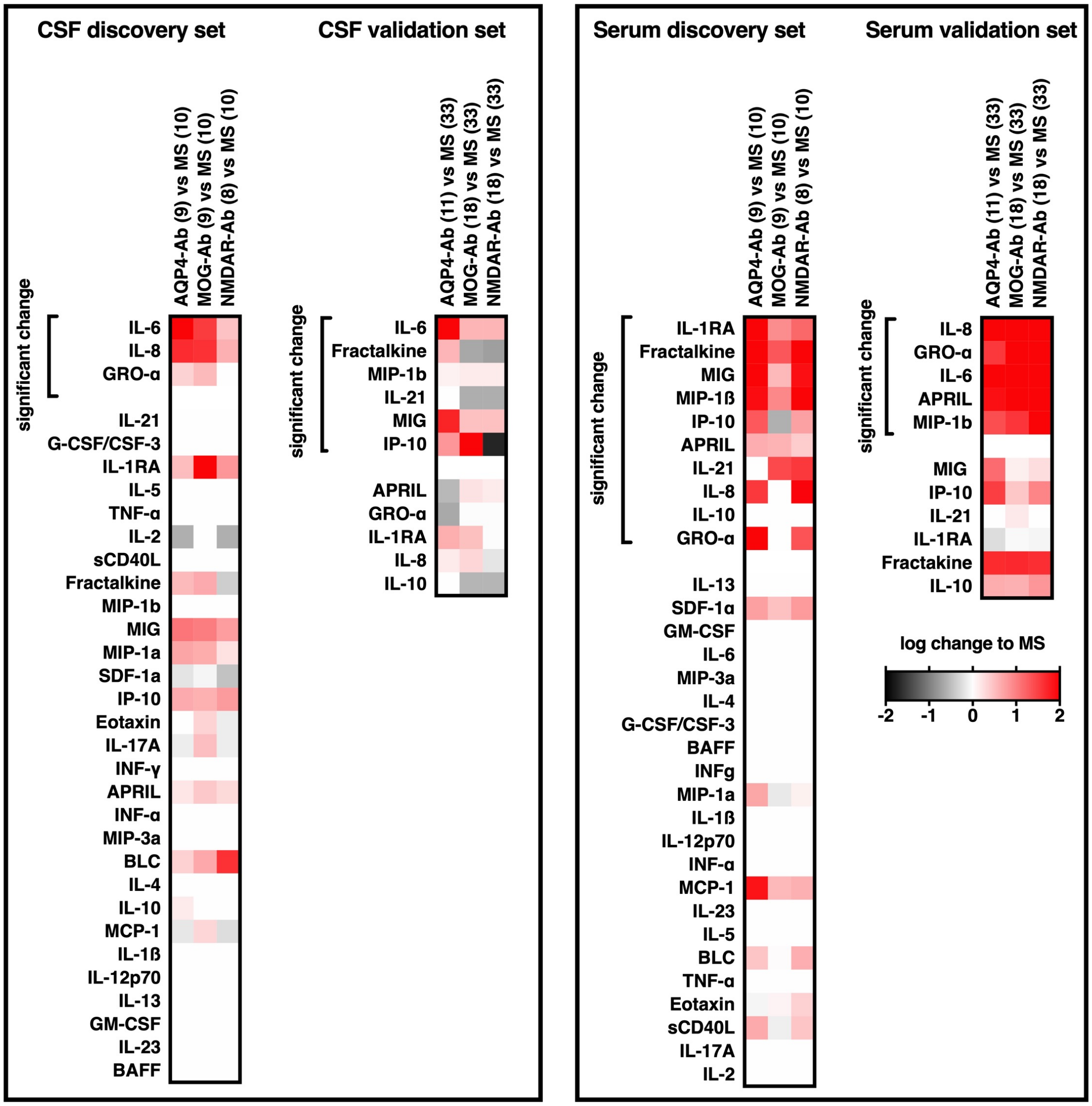

Supplementary Figure 1.

Cytokine and chemokine profile in CSF and serum of the discovery and validation set. The median log changes of median cytokine or chemokine levels compared to the MS group are shown in the heatmap. Cytokines and chemokines are organized according to their p-values. Significant changes in the CSF (AQP4-Ab and MOG-Ab versus MS) and serum (AQP4-Ab, MOG-Ab and NMDAR-Ab versus MS) are highlighted.
