# Supplementary material for: Distinct serum and cerebrospinal fluid cytokine and chemokine profiles in autoantibody-associated demyelinating diseases
Source: Mult Scler J Exp Transl Clin. 2019 May 15;5(2):2055217319848463. doi: 10.1177/2055217319848463 (PMC6537078; doi:10.1177/2055217319848463)
Supplement: Supplemental Material2 - Supplemental material for Distinct serum and cerebrospinal fluid cytokine and chemokine profiles in autoantibody-associated demyelinating diseases [file Supplemental_Material2.pdf]

Supplementary Table 1. Cytokines and chemokines of the discovery (all) and validation (bold) experiment.

Th1	Th2	Th17	Treg	Tfh	B cell-related	Others
IFN-γ	IL-4	**IL-6**	**IL-10**	SDF-1α (CXCL12)	**APRIL**	IL-1β
IL-2	IL-5	**IL-8 (CXCL8)**			BAFF	**IL-1RA**
IL-12p70	IL-13	IL-17A (CTLA-8)			BLC (CXCL13)	IFN-α
**IP-10 (CXCL10)**	Eotaxin	**IL-21**				CD40L
**MIG (CXCL9)**		IL-23				**GRO-α (KC/CXCL1)**
TNF-α		G-CSF (CSF-3)				MCP-1 (CCL2)
		GM-CSF				MIP-1α (CCL3)
						**MIP-1β (CCL4)**
						MIP-3α (CCL20)
						**Fractalkine (CX3CL1)**
