# Supplementary material for: Distinct serum and cerebrospinal fluid cytokine and chemokine profiles in autoantibody-associated demyelinating diseases
Source: Mult Scler J Exp Transl Clin. 2019 May 15;5(2):2055217319848463. doi: 10.1177/2055217319848463 (PMC6537078; doi:10.1177/2055217319848463)
Supplement: Supplemental Material3 - Supplemental material for Distinct serum and cerebrospinal fluid cytokine and chemokine profiles in autoantibody-associated demyelinating diseases [file Supplemental_Material3.pdf]

Supplementary Table 2. Clinical, demographic and immunological associations of increased CSF IL-6 levels in AQP4 and MOG autoantibody associated demyelinating diseases (without anti-NMDAR encephalitis).

	AQP4-Ab		MOG-Ab	
Parameters	CSF IL-6 levels < 17.6 pg/ml	CSF IL-6 levels ≥ 17.6 pg/ml	CSF IL-6 levels < 17.6 pg/ml	CSF IL-6 levels ≥ 17.6 pg/ml
Number of cases	8	12	12	15
MOG-Ab titre (1:) [1]	na	na	640 (320-10240)	1280 (160-81920)
Females	8/8 (100%)	11/12 (92%)	7/12 (58%)	6/15 (40%)
Age (years) [1]	51 (19-76)	52.5 (24-83)	27.5 (4-68)	21 (1-50)
Children	0/8 (0%)	0/12 (0%)	4/12 (33%)	7/15 (47%)
Disease duration (months)	1 (1-26)	0.5 (0-94)	43 (0-264)	0 (0-173)
Acute phase [2]	6/6 (100%)	9/9 (100%)	6/6 (100%)	7/7 (100%)
Clinical presentation [3]				
ON	2/7 (29%)	1/12 (8%)	4/11 (36%)	1/14 (7%)
TM	3/7 (43%)	6/12 (50%)	2/11 (18%)	7/14 (50%)
ON+TM	1/7 (14%)	4/12 (33%)	2/11 (18%)	2/14 (14%)
Brain stem	0/7 (0%)	1/12 (8%)		
Cerebral	1/7 (14%)	0/12 (0%)	3/11 (27%)	4/14 (29%)
Diagnosis NMOSD [4]	7/7 (100%)	12/12 (100%)	0/12 (0%)	2/15 (13%)
CSF cells [1]	10 (0-182)	7 (0-1758)	2 (0-11)	144 (2-192) *
CSF proteins (mg/dl) [1]	41 (30-499)	79 (30-141)	30 (19-35)	49 (30-124) *

Data are shown as [1] median (range); [2] data available from 28 patients; [3] data available from 44 patients; [4] data available from 46 patients. * statistically significant difference within the MOG-Ab subgroup at p<0.05 (Mann Whitney U test).
