# Supplementary material for: Distinct serum and cerebrospinal fluid cytokine and chemokine profiles in autoantibody-associated demyelinating diseases
Source: Mult Scler J Exp Transl Clin. 2019 May 15;5(2):2055217319848463. doi: 10.1177/2055217319848463 (PMC6537078; doi:10.1177/2055217319848463)
Supplement: Supplemental Material4 - Supplemental material for Distinct serum and cerebrospinal fluid cytokine and chemokine profiles in autoantibody-associated demyelinating diseases [file Supplemental_Material4.pdf]

Supplementary Table 3. Clinical, demographic and immunological associations of increased serum IL-8 levels in in AQP4 and MOG autoantibody associated demyelinating diseases (without anti-NMDAR encephalitis).

	AQP4-Ab		MOG-Ab	
Parameters	Serum IL-8 levels < 3.7 pg/ml	Serum IL-8 levels ≥ 3.7 pg/ml	Serum IL-8 levels < 3.7 pg/ml	Serum IL-8 levels ≥ 3.7 pg/ml
Number of cases	3	17	9	18
MOG-Ab titre (1:) [1]	na	na	480 (160-1280)	1280 (160-81920)
Females	3/3 (100%)	16/17 (94%)	6/9 (67%)	7/18 (39%)
Age (years) [1]	55 (52-62)	49.5 (19-83)	26 (4-51)	18.5 (1-68)
Children	0/3 (0%)	0/17 (0%)	2/9 (22%)	9/18 (50%)
Disease duration (months)	1 (0-94)	1 (0-27)	0 (0-264)	7 (0-156)
Acute phase [2]	3/3 (100%)	12/12 (100%)	8/8 (100%)	5/5 (100%)
Clinical presentation [3]				
ON	1/3 (33%)	2/16 (12.5%)	0/8 (0%)	5/17 (29%)
TM	2/3 (67%)	7/16 (44%)	5/8 (62.5%)	4/17 (23.5%)
ON+TM	0/3 (0%)	5/16 (31%)	1/8 (12.5%)	3/17 (18%)
Brain stem	0/3 (0%)	1/16 (6%)		
Cerebral	0/3 (0%)	1/16 (6%)	2/8 (25%)	5/17 (29%)
Diagnosis NMOSD [4]	3/3 (100%)	16/16 (100%)	0/9 (0%)	2/18 (11%)
CSF cells [1]	4 (1-1758)	10 (0-182)	48 (2-192)	2 (0-180)
CSF proteins (mg/dl) [1]	84 (32-141)	56 (30-499)	42 (30-120)	30 (19-124)

Data are shown as [1] median (range); [2] data available from 28 patients; [3] data available from 44 patients; [4] data available from 46 patients.
