# Supplementary material for: Distinct serum and cerebrospinal fluid cytokine and chemokine profiles in autoantibody-associated demyelinating diseases
Source: Mult Scler J Exp Transl Clin. 2019 May 15;5(2):2055217319848463. doi: 10.1177/2055217319848463 (PMC6537078; doi:10.1177/2055217319848463)
Supplement: Supplemental Material5 - Supplemental material for Distinct serum and cerebrospinal fluid cytokine and chemokine profiles in autoantibody-associated demyelinating diseases [file Supplemental_Material5.pdf]

Supplementary Table 4. Clinical, demographic and immunological associations of increased serum GRO-α levels in AQP4 and MOG autoantibody associated demyelinating diseases (without anti-NMDAR encephalitis).

	AQP4-Ab		MOG-Ab	
Parameters	Serum GRO-α levels < 7.2 pg/ml	Serum GRO-α levels ≥ 7.2 pg/ml	Serum GRO-α levels < 7.2 pg/ml	Serum GRO-α levels ≥ 7.2 pg/ml
Number of cases	8	12	8	19
MOG-Ab titre (1:) [1]	na	na	640 (160-1280)	1280 (160-81920)
Females	7/8 (87.5%)	12/12 (100%)	6/8 (75%)	7/19 (37%)
Age (years) [1]	59 (50-83)	48 (19-68)	25.5 (4-51)	16 (1-68)
Children	0/8 (0%)	0/12 (0%)	1/8 (12.5%)	10/19 (53%)
Disease duration (months)	0.5 (0-94)	13.5 (0-27)	39.5 (0-264)	0 (0-156)
Acute phase [2]	6/6 (100%)	9/9 (100%)	8/8 (100%)	5/5 (100%)
Clinical presentation [3]				
ON	2/8 (25%)	1/11 (9%)	1/7 (14%)	4/18 (22%)
TM	3/8 (37.5%)	6/11 (54.5%)	4/7 (57%)	5/18 (28%)
ON+TM	2/8 (25%)	3/11 (27%)	0/7 (0%)	4/18 (22%)
Brain stem	1/8 (12.5%)	0/11 (0%)		
Cerebral	0/8 (0%)	1/11 (9%)	2/7 (29%)	5/18 (28%)
Diagnosis NMOSD [4]	8/8 (100%)	11/11 (100%)	0/8 (0%)	2/19 (10.5%)
CSF cells [1]	5.5 (1-1758)	11 (0-182)	48 (2-192)	2 (0-180)
CSF proteins (mg/dl) [1]	77.5 (32-141)	54 (30-499)	42 (19-120)	30 (30-124)
Data are shown as [1] median (range); [2] data available from 28 patients; [3] data available from 44 patients; [4] data available from 46 patients.				
